# CRISPR Tackles Emerging Viral Pathogens

**DOI:** 10.3390/v13112157

**Published:** 2021-10-26

**Authors:** Emily N. Kirby, Byron Shue, Paul Q. Thomas, Michael R. Beard

**Affiliations:** 1 Research Centre for Infectious Diseases, Department of Molecular and Biomedical Sciences, School of Biological Sciences, The University of Adelaide, Adelaide 5005, Australia; emily.kirby@adelaide.edu.au (E.N.K.); byron.shue@adelaide.edu.au (B.S.); 2 Adelaide Medical School, The University of Adelaide, Adelaide 5000, Australia; paul.thomas@adelaide.edu.au; 3 Robinson Research Institute, The University of Adelaide, Adelaide 5006, Australia; 4 Genome Editing Program, South Australian Health & Medical Research Institute, North Terrace, Adelaide 5000, Australia

**Keywords:** CRISPR KO, CRISPRa, coronavirus, flavivirus, SARS-CoV-2, genome editing, viral life cycle, host factors, pro-viral, anti-viral

## Abstract

Understanding the dynamic relationship between viral pathogens and cellular host factors is critical to furthering our knowledge of viral replication, disease mechanisms and development of anti-viral therapeutics. CRISPR genome editing technology has enhanced this understanding, by allowing identification of pro-viral and anti-viral cellular host factors for a wide range of viruses, most recently the cause of the COVID-19 pandemic, SARS-CoV-2. This review will discuss how CRISPR knockout and CRISPR activation genome-wide screening methods are a robust tool to investigate the viral life cycle and how other class 2 CRISPR systems are being repurposed for diagnostics.

## 1. Introduction

Viruses are dependent on the cellular environment for their life cycles and the dynamic relationship between a virus and its host is ever evolving in the hope to maximise viral fitness and dissemination to the next host. This has been evident in the ongoing Sudden Acute Respiratory Syndrome Coronavirus-2 (SARS-CoV-2) pandemic in which the evolution of a virus can be observed in real time through molecular methods to track viral evolution. At the time of writing, SARS-CoV-2 is responsible for approximately 177M infections globally, with 3.8M deaths [1]. However, this is not the first time that viral pathogens have challenged humanity with outbreaks of SARS-CoV (2002), MERS-CoV (2012), Zika Virus (2015), Spanish Flu (1918) and the constant threat from HIV, Dengue Virus and many more [2,3,4,5,6]. 

Due to the relatively small size of viral genomes, viruses rely heavily on the cellular environment for their replication and dissemination. This can include co-opting host proteins and machinery for genome replication, translation and silencing of host innate and adaptive immune responses, all known as pro-viral host factors [7,8,9]. In contrast, the host recognises viral infection through a complex array of proteins collectively termed pattern-recognition receptors (PRRs), to orchestrate expression of anti-viral-effector molecules to inhibit viral replication and elicit immune activation [10,11]. Thus, our knowledge of the viral host relationship is crucial for our understanding of viral replication dynamics and ultimately in vaccine and therapeutic design.

To identify host proteins that are co-opted by viruses throughout their life cycles, several platforms have been used such as novel and established compound screening and functional genomics via technologies such as small interfering RNAs (siRNA) and short hairpin RNA (shRNA) that reduce gene expression and mediate the viral replication capacity in response [12,13,14,15]. However, it was the adaptation of CRISPR-Cas technology to in vitro and in vivo models that allowed for an efficient method to induce complete knockout of gene expression. CRISPR also allows for simultaneous functional characterisation of most genes within the genome, allowing researchers to generate vast amounts of data within a few weeks. 

Furthermore, CRISPR-based approaches have also been used to target viral genomes directly with the aim of viral elimination. Examples include Hepatitis B Virus (HBV) and Human Immunodeficiency Virus (HIV), and while somewhat successful in in vivo and in vitro models, this novel strategy is still in the development phase [16,17,18,19,20,21,22]

Consequently, CRISPR is a highly used tool in virology to understand the molecular interactions between viruses and their host. Hence, this review will focus on how the various CRISPR systems have been used to identify novel interactions and how future advancements could be used as therapeutics and diagnostic tools.

## 2. Origins of CRISPR Genome Editing Technology

CRISPR is a prokaryotic defence mechanism against invading bacteriophage [23], and comprises two classes that can be further subdivided into types I–VI, dependent on the number of CRISPR-associated (*Cas*) nuclease proteins utilised and their loci arrangement [24,25]. Class 1, found in archaea and some bacterial species, is composed of types I, III and IV. They are distinct from class 2 due to their complex use of multiple effector *Cas* proteins, anywhere from 4 to 7 subunits, and are therefore not widely used in genome editing [24,26].

Class 2 is composed of types II, V and VI, with type II harnessed from *Streptococcus pyogenes* commonly associated with CRISPR genome editing [25]. Type II utilises the *Cas9* endonuclease, where *Cas9* is guided by a chimeric crRNA:tracrRNA, called a guide RNA (gRNA) (Table 1). The first crRNA contains a 17–20 bp RNA sequence complementary to the target that is upstream of a protospacer adjacent motif (PAM) sequence. PAM sequences are required for *Cas* to discriminate self from non-self as they are only found in the invading bacteriophage genome. For commonly utilised type II systems incorporating *Cas9* from *Streptococcus pyogenes*, the PAM sequence is 5′NGG. The trans-activating crRNA (tracrRNA) has multiple roles in maturation of the crRNA from pre-crRNA in conjunction with RNase III [27,28] and acts as a scaffold for *Cas9* binding [28,29,30]. Binding of the gRNA to *Cas9* induces conformational change in *Cas9* activating endonuclease activity. Upon binding of the gRNA to the target and identification of the PAM sequence, the HNH domain of *Cas9* cleaves the complementary bacteriophage DNA strand, while the RuvC cleaves the non-complementary strand [28,31]. Cleavage produces a double-stranded break (dsb), rendering the invading phage unable to replicate and induce bacterial cell death [23,32]. 

The significant advance in genome editing technology arose when CRISPR was adapted to knockout gene expression in eukaryotic in vitro models [28]. The efficiency and simplicity of CRISPR to induce dsb in the genome far surpassed traditional technologies such as TALENS and ZFNs [33,34]. Subsequently, the rapid development of CRISPR technology now allows researchers to knockout a single gene within a genome in vitro and in vivo, allowing advanced investigation into the role of a protein in molecular pathways and organism physiology.

Most recently, the class 2 system has expanded to include type V, which differs in use of the endonuclease *Cas12,* (Table 1) [24,26]. While both *Cas9* and *Cas12* share a RuvC domain, *Cas12* lacks the HNH domain which is replaced with an uncharacterised Nuc domain [35,36]. Additionally, type V can be further subdivided into groups A–E, with extensive characterisation of groups A and B. The Group V-A endonuclease, previously known as Cpf1, does not have a requirement for a tracrRNA, utilising only a crRNA that can activate *Cas12* conformational change. Maturation of the crRNA occurs upon binding with *Cas12,* allowing *Cas12* to cleave the pre-crRNA [37]. One similarity across all subtypes is the requirement for a 5′ TTN PAM sequence, making *Cas12* a potentially suitable alternative for genome editing of T-rich genomic sequences [35,36,37]. *Cas12* can also indiscriminately cleave single-stranded DNA upon recognition of a target sequence, complementary to the guide RNA and as such is being repurposed for diagnostic use [38,39,40].

Research into type VI *Cas13* systems is also rapidly expanding due to their unusual ability to target and cleave RNA instead of DNA, due to the presence of 2 Higher Eukaryotes and Prokaryotes Nucleotide (HEPN)—binding domains with ribonuclease activity (Table 1) [25,41]. *Cas13* enzymes utilise only a crRNA complementary to the target RNA, which allows for RNA:RNA hybridisation. Advantageously, *Cas13* does not have a requirement for a PAM sequence, increasing the flexibility of crRNA target sites; however, some species of *Cas13* do prefer a protospacer flanking site, which incorporates either an additional A, U or C (species dependent) at the 3′ end [42,43,44]. *Cas13* also displays collateral cleavage activity, where, upon recognition, *Cas13* indiscriminately cleaves single-stranded RNA transcripts, which is believed to part of a bacterial programmed cell death response to infection [42,45,46]. However, this collateral activity is not present in mammalian cells and so *Cas13* is being repurposed as an alternative to siRNA- and shRNA-mediated gene knockdown, RNA editing and in infectious disease diagnostic testing [44,47,48,49,50,51]. Additionally, inactivation of the HEPN domains leads to inhibition of RNA cleavage but allows *Cas13* to retain RNA binding activity and this “dead *Cas13*” is being optimised for use in live-cell imaging of RNA transcript movement in in vitro model systems [52].

Collectively, the class 2 CRISPR systems are now embedded as a key component in various lines of investigation, including genome editing for characterisation of a specific gene of interest, development of repair mechanisms for genetic inheritable diseases, RNA tracking and novel diagnostics. In addition to CRISPR targeting a specific nucleic acid sequence, it is now possible to target all known genes of any species through the development of CRISPR *Cas9* screening libraries, that contain a pool of guide RNAs with the intent to target all genes for either gene knockout or activation of gene expression. Briefly, CRISPR activation (CRISPRa) is a modification of the more well-known CRISPR knockout (CRISPR_KO_), where it uses *Cas9* that has lost its endonuclease activity, but still retains its sgRNA binding capacity. Further details on the CRISPRa system will discussed later in this review.

Given the importance of host proteins in the viral life cycle, these genome-wide CRISPR libraries are being utilised to understand this dynamic relationship, enabling the identification of pro- and anti-viral host factors. This provides insight into the replication strategies of pathogenic viruses, with the potential for future development of therapeutics.

**Table 1:** Summary of commonly used class 2 CRISPR systems and their uses in virology research and diagnostics.

		Nuclease Domains	PAM	Substrate	Cleavage	Collateral Cleavage?	Use in Virus Research
*Cas9*	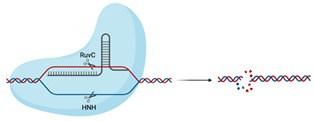	RuvC, HNH	5′-NGG-3′	dsDNA	Blunt ends	No	Utilised in numerous genome-wide and target-specific CRISPR screens to identify and characterise the relationship between cellular host factors and viruses (refer to Table 2) Used in studies aiming to inactivate integrated viral DNA that results in chronic infection (e.g., HBV) [53]
*Cas12*	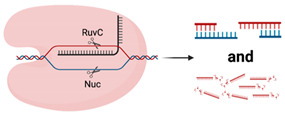	RuvC, Nuc	5′-TTTN-3′	dsDNA	5′ staggered overhang of 5 bp	Yes—ssDNA Not in mammalian or plant culture	Forms the basis of the DETECTR diagnostic method used for detection of viral nucleic acids (e.g., SARS-CoV-2) [40] Potential alternative for genome-wide screens due to alternative PAM sequence requirements, allowing potential to target T’-rich gene sequences [35]
*Cas13*	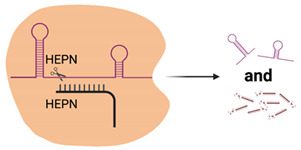	2× HEPN	Subspecies and culture model dependent. Preference for 5′ “Protospacer Flanking Sequence”	ssRNA	Cleavage at uracil	Yes—ssRNA Not in mammalian or plant culture	Forms the basis of the SHERLOCK diagnostic method for detection of viral nucleic acids (e.g., SARS-CoV-2) [51] Shown in cell culture models to cleave viral RNA and inhibit replication [50] Development of dCas13 as a method to track viral RNA movement in the cell throughout viral replication [52]

## 3. CRISPR Knockout Screening

Prior to the establishment of CRISPR genome editing technology as a staple molecular technique, screening to identify host factors critical to viral replication and inhibition included haploid genetic screens and RNA interference (RNAi).

Haploid genetic screens, as the name infers, use haploid cells (single allele for each gene) with gene knockout mediated by lentiviruses/retrovirus or transposon-mediated insertional mutagenesis. Haploid screens are advantageous as there is complete gene inactivation, unlike in cells of diploid origin, where there is the risk that is the second allele is not mutated and remains functional. However, haploid screens are limited to cell type availability, which may not truly represent the viruses natural cell reservoir. In all, haploid screens have been successful in identifying of a number of pro-viral factors, and have enhanced our understanding of viral host dynamics [54,55,56,57].

Another method to identify and characterise the role of host factors in the viral life cycle is short hairpin RNA (shRNA) or small interfering RNA (siRNA) screens to inhibit endogenous gene expression [58,59] as they can be efficiently introduced into cells and utilise the endogenous RNA degradation proteins Drosha and Dicer [60]. Numerous shRNA/siRNA screens have been performed in the past and identified a number of cellular proteins such as host kinases, cellular receptors, transcription factors and transporter proteins that are critical to viral replication [14,15,61,62,63,64]. However, little overlap exists between top hits of independent screens, primarily driven by varying siRNA sequences for each gene, differential off-target effects and knockdown efficiencies [58]. As such, silencing efficiency of several siRNAs targeting the same gene can vary significantly. Residual activity due to incomplete gene knockdown also makes it difficult to ascertain the degree of impact the host factor in question has on viral replication. Observation of high off-target rates observed in RNAi screens may also be attributed to (i) incomplete binding of siRNA onto the 3′UTR of mRNA which can facilitate miRNA-like inhibition, resulting in down-regulation of non-target genes and (ii) during the delivery of siRNA into the cell, the innate immune response can be activated by recognition of RNA in the endosome by Toll-Like Receptors [65]. This can lead to different phenotypes observed due to the activation of immune pathways and production of inflammatory cytokines [65,66].

However, most recently with the explosion of CRISPR technology, CRISPR_KO_ screening is now the preferred method as it avoids differential off-target effects and associated knockdown efficiency issues that are often encountered when using siRNAs. As CRISPR_KO_ can induce a complete sustained reduction in target gene expression, this has expanded the potential to identify novel host factors that are critical for viral replication that may have been otherwise gone undetected in siRNA screening platforms.

**Figure 1:**
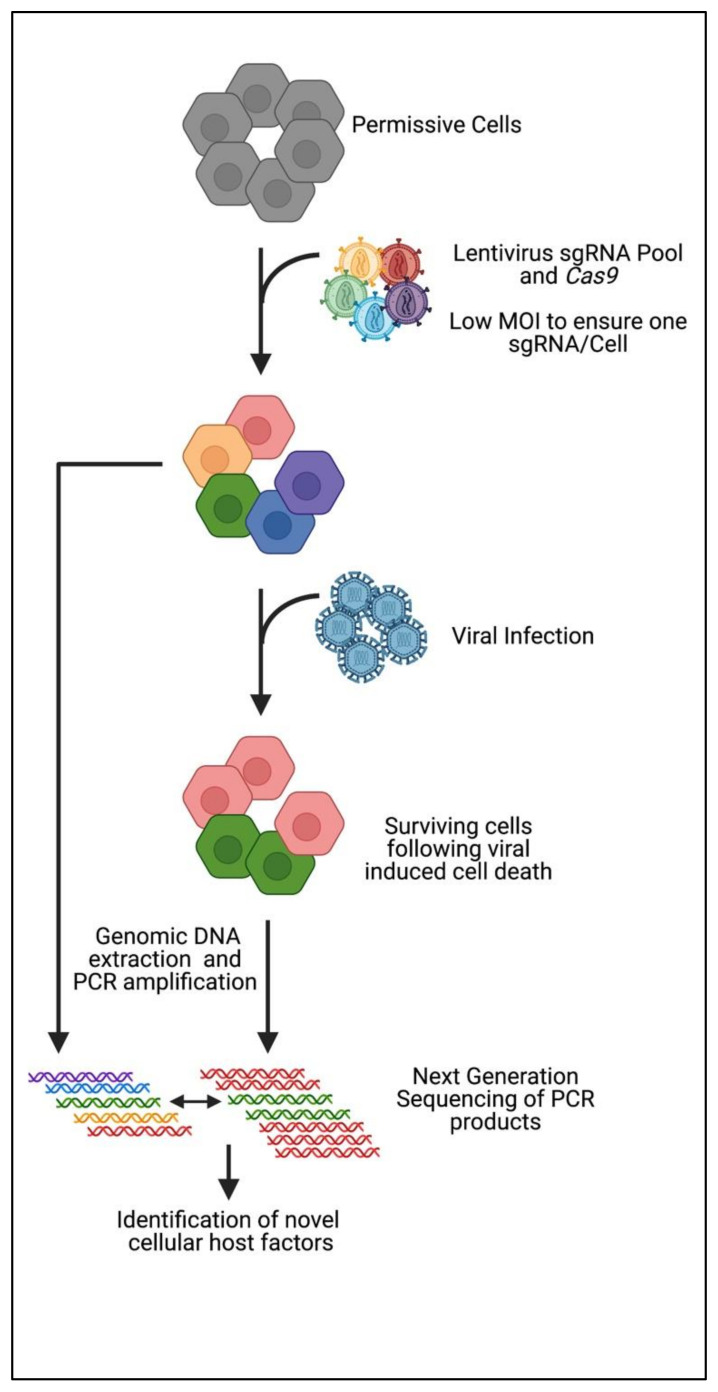
Workflow of the genome-wide CRISPR_KO_ screening process for the identification of novel pro-viral host factors key for viral life cycles.

**Figure 2:**
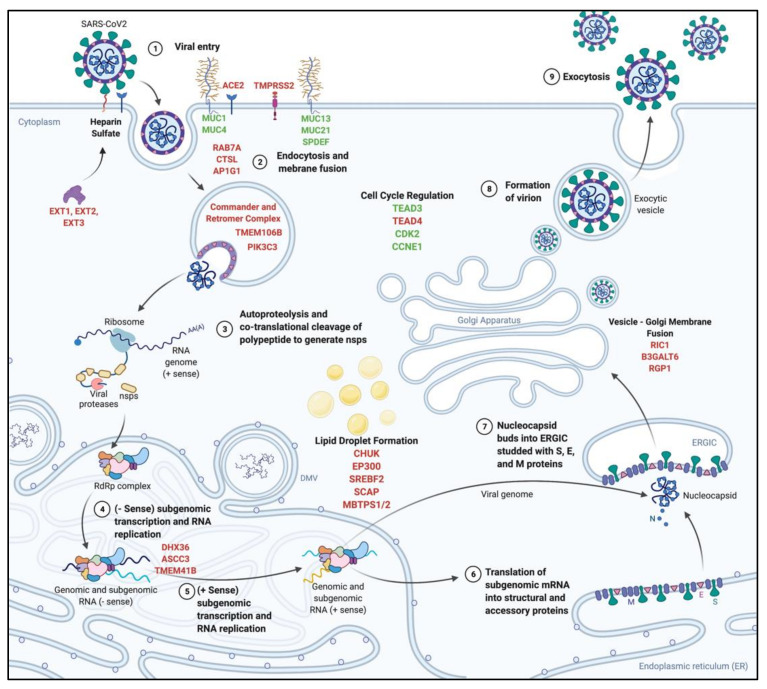
Schematic of SARS-CoV-2 replication highlighting putative pro-viral (identified from CRISPR_KO_ screens—red) and anti-viral (identified from CRISPRa screens—green) host factors. Viral and host protein interactions and cellular localisation are indicated based on known host factor localisation; however, this warrants further validation and characterisation.

In general, genome-wide CRISPR_KO_ screens to date rely on the cytopathic (CPE) nature of viral infection to select for cells that survive following lentiviral transduction of sgRNAs (Figure 1). Surviving cells are therefore not permissive to viral infection as a result of CRISPR-mediated KO of a pro-viral host factor. Following expansion of surviving cells, genomic DNA is subjected to PCR amplification of the integrated sgRNA sequences and NGS used to determine the relative enrichment of specific guide sequences indicating possible key cellular proteins involved in the viral life cycle. As such, several CRISPR_KO_ screens have been performed to identify key cell factors in significant pathogens of the *Flavivirdae* family (Zika, Dengue, West Nile, Yellow Fever, and Hepatitis C), Influenza A, Epstein–Barr Virus, Norovirus, Ebola Virus and Human Immunodeficiency Virus. These screens have been reviewed previously and will not be discussed further; however, the key findings are summarised previously in Table 2. We will therefore focus on recent CRISPR_KO_ screens that have yet to be reviewed and how this has impacted on our understanding of the interaction between host factors and viruses. We will discuss SARS-CoV-2, the coronavirus responsible for the COVID19 pandemic, with several putative host factors and their inferred localisation of activity highlighted in Figure 2, and Zika Virus.

### The Coronaviruses

Coronaviruses impose a significant health and economic burden. Of the seven known human coronaviruses (CoV), four are endemic (HCoV-229E, -OC43, -NL63 and -HKU1) and one pandemic-CoV, SARS-CoV-2, currently in the human population. Two additional human CoV respiratory pathogens, SARS-CoV and MERS-CoV, have also emerged in recent times [94,95]. SARS-CoV was essentially controlled through public health measures while MERS-CoV still circulates within camels [2]. In contrast to SARS-CoV and MERS, SARS-CoV-2 has for many countries evaded public health measures and is now causing havoc on a global scale. As such, there is now significant focus on understanding SARS-CoV-2 pathogenesis, replication kinetics and the dynamics of the virus–host relationship.

The coronaviruses have exceptionally large single-stranded RNA genomes of approximately 30 kB with a 5′-cap and 3′-poly-A tail. Following receptor-mediated viral entry (i.e., ACE2 for SARS-CoV-2) the genome is release into the cytoplasm and directly translated by host ribosomes. The 5′ two-thirds of the genome encodes two polyproteins, pp1a and pp1ab with the latter generated by ribosomal frameshifting. The polyprotein is cleaved into 16 non-structural proteins including the RNA-dependent RNA polymerase. The 3′ third encodes four structural proteins (S, E, M and N) and a set of accessory proteins that can interfere with host innate responses. A summary of the coronavirus life cycle is outlined in Figure 2 and reviewed in [96]. At all points in the replication cycle, coronaviruses rely on cellular host factors and identification of these interactions represents sites that can be potentially exploited for therapeutic gain. While there are differences between the circulating common cold coronaviruses and SARS-CoV-2, many of the fundamental replication processes are similar and as such the common cold and animal coronaviruses such as Mouse Hepatitis Virus (MHV) and Infectious Bronchitis Virus (IBV) have been used as surrogates for the study of the more pathogenic human beta-coronaviruses such as SARS and MERS that are restricted to PC3 laboratory conditions [97].

The cytopathic nature of the coronaviruses and specifically SARS-CoV-2 provides a well-defined phenotypic endpoint for CRISPR screens and a number of independent CRISPR_ko_ screens [84,86,87,88,89,90,93] have identified several novel host factors essential for replication (Table 1). Not unexpected and common hits in all screens include the entry receptor, ACE2 and Cathepsin L1 (CTSL1), a proteinase involved in cleavage of the S1 subunit of SARS-CoV-2 spike protein upon entry, enabling membrane fusion [84,86,87,88,89,90]. Screens performed in the lung carcinoma cell line A549, identified an enrichment of hits associated with the Retromer and Commander complexes [86,90]. Both these large protein complexes are involved in the recycling of transmembrane proteins and receptors from endosomes by interaction with the trans-Golgi network to maintain cell surface expression levels following endocytosis [98,99]. These complexes may provide a mechanism for internalisation and/or uncoating of the genome from the virion or in later stages of the life cycle such as trafficking to Golgi bodies for exocytosis; however, further investigation is required to determine the role that these complexes play at various points in the SARS-coV-2 life cycle.

Highlighting the intrinsic nature of cell type-specific pro-viral host factors, screens performed in different cell types have uncovered enrichment of cell-specific host factors. This is highlighted in screens performed using the hepatocellular carcinoma cell line Huh-7, which expresses both ACE2 and TMPRSS2 receptors. Transmembrane protein 106B (TMEM106B) is a lysosomal transmembrane protein associated with lysosome trafficking and activity in motor neurons and dendritic cells and is the cause of frontotemporal dementia in patients with large deletions [100,101]. CRISPR KO of TMEM106B in Huh-7 cells abrogates SARS-CoV-2 replication that was further confirmed in cell lines of lung origin and primary bronchial epithelial cells [84,87]. Given the critical role of lysosomes in SARS-CoV-2 infection, it is possible that TMEM106B is a mediator of viral membrane fusion with the endosome membrane, to allow genome release into the cytosol.

Exostosin 1 and 2 (EXT1, EXT2), which together are the endoplasmic reticulum-resident type II transmembrane glycosyltransferase proteins [84,87,88] have also been identified as essential cellular proteins involved in SARS-CoV-2 replication. EXT1 and 2 are responsible for extension of heparan sulfate chains, which are proteoglycans expressed on the cell surface as part of the extracellular matrix [102,103]. Interestingly, heparan sulfate has been shown to be essential for mediating interaction of SARS-CoV-2 Spike protein with the ACE2 receptor. Heparan sulfate directly interacts with the positively charged S1 domain of the Spike protein, inducing a conformational change to an “open” state, suggesting it is a co-factor for entry [104,105]. Heparan sulfate also mediates entry of Herpes Simplex Virus, Dengue Virus and Human Immunodeficiency Virus [106,107,108]. Additional screens performed comparing SARS-CoV2 with other closely related *Betacoronaviruses* (HCoV-OC43) and more distant *Alphacoronaviruses* (HCoV-NL63 and HCoV-229E), showed again the importance of heparan sulfate in entry of *betacoronaviruses* as knockout of EXT1-3 inhibited replication of HCoV-OC43, but not NL63 and 229E [88]. Given that NL63 spike protein mediates endocytosis via ACE2, and is associated with heparan sulfate binding, it is possible that some *alphacoronaviruses* do not critically rely on heparan sulfate for cell binding and endocytosis but may mediate this via another receptor [109,110]. This difference in the host-viral protein networks of two related viruses may have gone unnoticed if not for the power of CRISPR, showing that it is highly beneficial tool for studying virus–host interactions and development of virus-specific anti-virals.

Variation in enrichment of host factors can arise in response to different cell lines used. As example, Calu-3 and A549 cells, both of which are of lung epithelial origin, revealed ACE2 as the top hit; however, the AP-1 complex subunit gamma-1 (AP1G1), a clatherin-adaptor protein expressed as part of the trans-golgi network, is significantly enriched in Calu-3s but not A549 cells. While AP1G1 is potentially mediating the role of either virus endocytosis or egress in Calu-3s, it is possible that this is not the case in A549s, which may use other host factors for these processes [91].

CRISPR can also be used to identify pan-viral host factors, such as TMEM41B, another transmembrane protein that is associated with autophagy, wherein by TMEM41B knockout reduces the mobilisation of lipids from lipid droplets to mitochondria and autophagosome formation [88,92,93,111,112,113]. TMEM41B was also identified as a host factor for the *flaviviruses* ZIKV, YFV, DENV and WNV, a family of +ssRNA viruses that also induce membrane rearrangements to form replication complexes much like *coronaviruses.* In *flaviviruses,* it was found that TMEM41B localises with NS4A and NS4B at replication complexes, mediating ER rearrangement [114]. The importance of TMEM41B was established in *coronaviruses,* where knockout of TMEM41B in 3 independent screens significantly inhibited the replication of SARS-CoV2, OC43, NL63 and 229E [88], MERS-CoV and 229E [92] and 229E alone [93], but was rescued upon re-complementation [88,93]. SARS-CoV2 infection showed TMEM41B localisation to cytosolic sites, likely ER membranes [88]. Further studies with HCoV-229E show that TMEM41B does not co-localise with any non-structural proteins at sites of replication complex formation but does alter the availability of free cholesterols in the cytoplasm by sequestering lipids into enlarged lipid droplets and preventing replication complex formation [93]. However, this observation is still to be confirmed for SARS-CoV-2 and other related *coronaviruses.*

Strikingly, only CRISPR_ko_ screens that use Calu-3 cells show enrichment of sgRNAs for the SARS-CoV-2 spike priming cellular serine protease, TMPRSS2, that is critical for SARS-CoV and SARS-CoV-2 entry [115,116,117,118,119]. TMPRSS2 mediates the cleavage of S1/S2 and S2 prime sites of both SARS, resulting in the production of several smaller fragments, priming the S protein for interaction with ACE2 and membrane fusion [116]. TMPRSS2 has also been shown to be critical for mediating hemagglutinin cleavage of Influenza [120], in addition to a role in mediating downstream gene expression in cells of prostate carcinoma origin [121,122]; however, its specific function in healthy tissue remains poorly characterised. It is possible that TMPRSS2 may mediate a role in cell survival, with CRISPR_KO_ inducing cell death that may explain why the sgRNAs where not highly represented in surviving cells of differing origin. This is a limitation of CRISPR_KO_ screens, in that knockout of host survival factors cannot be identified in a screen that utilises cell survival in the face of cytopathic virus replication as a phenotypic screening endpoint. Consequently, cell factors that are key for cell viability and important for SARS-CoV-2 life cycle may not be identified. Alternatively, a yet unidentified cell serine protease may allow SARS-CoV-2 spike protein modification and cell entry that is expressed significantly in other cells lines, that may not be present in Calu-3s. This redundancy may explain why TMPRSS2 is critical for these cells and not others.

An interesting observation from all CRISPR_KO_ screens discussed above is the extreme diversity of hits across the various cell lines used. This could be attributed to a host of reasons including different experimental protocols, different CRISPR_KO_ platforms and as mentioned above, different cell types. Additionally, continuous culture of viruses in specific cell lines will drive adaptation for the use of cell-dependent host factors. Consequently, physiological cell types preferably of primary origin and low passage virus will be key to identifying physiologically relevant pro-viral host factors that may inform anti-viral therapeutics.

CRISPR_KO_ also highlights an ability to distinguish pro-viral host factors across differing virus families, exemplified by the lack of similarity between the hits identified for *flaviviruses* and *coronaviruses*. This is especially so, given that both viral families are +ssRNA genomes, replicate in the cytosol, form ER membrane replication complexes and egress via the trans-golgi network [123]. Thus, CRISPR_KO_ shows that it is powerful enough to delineate these critical differences in the replication of similar, but distinct viruses and allows development of a deeper understanding of the complex nature of the viral life cycle and the consequential disease pathologies.

## 4. CRISPR Activation Screening

The ability to manipulate CRISPR componentry has most recently resulted in generation of gene activation (CRISPRa) systems that enhance targeted gene expression to a static upper limit, indicative of maximal endogenous expression. CRISPRa uses a modified *Cas9* protein in which mutations are introduced into the critical RuvC (D10A) and HNH (H840A) domains rendering the endonuclease activity “dead” and is hence termed “d*Cas9*” (Table 3). As such, d*Cas9* is unable to cleave DNA but still retains the capacity to interact with sgRNA and bind to targeted DNA sites. In contrast to CRISPR_ko_ where sgRNA target exon coding regions, sgRNA in CRISPRa are complementary to the proximal promoter, ~100–500 bp upstream of a transcriptional start site (TSS). Transcription activators (contain a DNA binding domain and an activation domain) fused to d*Cas9* are required for recruitment of canonical transcription factors that regulate transcriptional processes such as unwinding of DNA from the chromatin or formation of the scaffolding required for RNA polymerase recruitment. RNA pol can then transcribe the gene of interest, driving mRNA transcription and functional protein expression. This provides the benefit of increased gene expression from the endogenous promoter, allowing investigation of gene function of all transcript variants in contrast to traditional cDNA overexpression strategies.

Commercially, several CRISPRa systems are available (refer to Table 3), each utilising a unique combination of transcriptional activators to enable gene activation.

### 4.1. CRISPR-VPR

The CRISPR VP64-p65-Rta system was one of the first modified CRISPRa systems in which *dCas9* was fused at its C-terminus with the transcriptional activator VP64, a tetramer of the Herpes Simplex Virus (HSV) VP16 protein that starts early gene transcription by recruitment of host transcriptional machinery. Additionally, a subunit of NF-κβ, p65, and Replication and Transcription Activator (RTA) of gamma herpesviruses, are fused at the C-terminus to VP64. The presence of three transcriptional activators is enough to drive recruitment of transcriptional machinery at the directed sgRNA binding site and induce gene transcription greater than dCas9-VP64 alone [124].

### 4.2. CRISPR-Synergistic Activation Mediator (SAM)

CRISPR-SAM also uses dCas9-VP64; however, the gamma herpesviruses RTA is not involved and p65 is not fused directly to VP64, rather it is found as part of a transcriptional helper complex including Heat Shock Factor 1 (HSF1) and the Bacteriophage MS2 coat protein that acts a linker to recruit the complex to dCas9. A modified sgRNA stem–loop structure incorporates 2× MS2 RNA aptamers that can be recognised by the MS2 coat protein complex linked to VP64 and HSF1. This allows the sgRNA in association with dCas9-VP64 to recruit the helper complex to the target promoter to enable gene transcription [125].

### 4.3. CRISPR-SunTag

In contrast to CRISPR-SAM outlined above, the CRISPR-SunTag system uses a *dCas9*-VP64 fusion, in which the yeast master transcriptional regulator, GCN4, in the form of a repeating polypeptide is fused to the C-terminus of *dCas9.* This peptide, named SunTag, is the target of small-chain-variable fragment (ScFv) antibodies raised against GCN4, that is fused to VP64 and GFP, with GFP enabling FACS selection of transcriptionally activated cells. Depending on the iteration of the SunTag system used this allows recruitment of between 10 and 24 VP64 proteins to the target promoter, enabling gene transcription [127,128].

Researchers have been quick to appreciate the value of the CRISPRa screens as a tool to identify novel anti-viral host restriction factors with published reports of activation screens being performed for Influenza A (H1N1 PR8) and Murine Norovirus (CW3 and CR6) and most recently for SARS-CoV-2 and ZIKV. The outcomes of these screes for IAV and MNoV have been reviewed elsewhere and are summarised in Table 1, and hence, in this review, we focus on the most recent ZIKV and SARS-CoV-2 screens.

### 4.4. Zika Virus

ZIKV has a positive-sense, single-stranded RNA genome and is a member of the Flaviviridae family. ZIKV is best known for its association with the 2015–2016 outbreak of South America, coinciding with the Rio Olympic Games [3,129,130].While not associated with a high mortality, ZIKV infection of pregnant women resulted in an increase in the number of children born with neurological disorders such as microcephaly, revealing ZIKV as the first *flavivirus* with the ability to vertically transmit from mother to child [130,131,132]. To identify critical anti-viral host restriction factors that could be further explored to understand ZIKV infection and pathogenesis, CRISPRa screening using the powerful genome-wide CRISPR SAM system was employed. Designed by Feng Zhang of Massachusetts Institute of Technology, the lentiSAMv2 library containing approximately 3 sgRNA per gene (to a total of ~113.00 sgRNA) was used to activate gene expression prior to cells being infected with ZIKV with surviving cells deemed to express a host restriction factor. sgRNAs for Interferon-Inducible Protein 6 (IFI6) and Interferon Lambda 2 (IFN-λ2) were highly enriched and further identified as potent inhibitors of ZIKV infection in Huh7 cells [73]. IFI6 has been associated with regulation of mitochondrial reactive oxygen species (mtROS) levels and cancer metastasis [133], DNA replication stress in melanoma [134] and inhibition of apoptosis at the mitochondria by regulation of caspase, Bcl-2 and Bax expression during DENV infection [135] although its role as a viral restriction factor was unclear. The use of IFI6 overexpression in Huh7 cells and subsequent infection with ZIKV revealed no impact on polyprotein translation, but a significant reduction in dsRNA abundance, a marker of replication complex formation. This suggests that IFI6 inhibits replication through a defect in replication complex formation at the ER [73]. Interestingly, a role for IFI6 as a host anti-viral factor was confirmed using a genome-wide CRISPR_KO_ screen to identify interferon induced host restriction factors that impact flavivirus replication [80]. While several canonical ISGs and members of innate immune activation pathways were identified, IFI6 was highly enriched in multiple screens. Further analysis using ectopic and endogenous IFI6 expression revealed that IFI6 was predominantly localised to the ER in contrast to its previously reported localisation to the mitochondria [136,137,138]. IFI6 was not directly anti-viral; however, through its interaction with the ER-resident heat shock protein 70 chaperone BiP (for stabilisation), it facilitated inhibition of ER membrane invaginations and formation of the viral replication complex. These independent studies highlight the capacity of different CRISPR screening strategies to identify similar and unique host factors involved in viral replication and/or restriction.

### 4.5. SARS-CoV-2

The ability of SARS-CoV-2 to readily replicate in culture and cause a cytopathic effect suggests that like CRISPR_KO_, CRISPRa has the potential to identify novel anti-viral host restriction factors and studies are now emerging. Using SARS-CoV-2 infected lung epithelial cells (Calu-3) [85], coupled with a CRISPRa (Calabrese library) screen identified enrichment of a number of sgRNAs corresponding to cell survival with the greatest enrichment being for Transcriptional Enhancer Factor TEF-5 (TEAD3). TEAD3 is activated downstream of the of the Hippo signalling complex, responsible for activating cell proliferation and regulation of organ size [139,140]. While it is not immediately apparent as to the role of TEAD3 anti-SARS-CoV-2 restriction, it is possible that it may interact with one of the many SARS-CoV-2 proteins or that it results in transcriptional gene expression changes that are currently uncharacterised. Interestingly, the closely related SARS-CoV induces cell cycle arrest and inhibition of apoptosis via the Nucleocapsid, NSP2, NSP3 and NSP15 proteins promoting an environment that is considered optimal for viral replication [141,142]. It is not inconceivable that expression of TEAD3 could promote an environment of cell proliferation, that negatively impacts SARS-CoV replication and enhanced cell survival.

CRISPRa genome-wide screens have also revealed enrichment for sgRNAs representing the known anti-viral Mucin family members, MUC1, -4, -13 and -21 [85,91]. Mucins are a family of O-glycosylated glycoproteins that are either membrane bound or secreted to form mucosa membranes. In the case of MUC1, -4, -13 and -21, all are membrane tethered and can exert anti-Influenza activity by steric hinderance of IAV hemagglutinin binding to sialic acid receptors and thus inhibition of entry. Infection of Calu-3 cells expressing ectopic MUC4 with a vesicular stomatitis virus (VSV) pseudovirus, expressing SARS-CoV-2 spike protein, revealed a significant reduction in cell entry. However, treatment of Calu-3 cells with the bacterial protease StcE, specific for mucins, reversed this phenotype while individual CRISPRa activation of MUC1, -4 and -21 was also anti-viral for the coronaviruses MERS-CoV and HCoV-229E. However, the importance of mucins in a physiological setting is unclear given that SARS-CoV-2 infection increases MUC1, -4, -13 and -21 in human and mouse lung tissue as determined by RNA Seq. This would suggest that like IAV, mucins inhibit entry of the virus, in this case preventing spike-mediated endocytosis. Whether this be by steric hinderance by blocking access to the ACE2 receptor or to other essential co-receptors that mediate spike conformational change (e.g., Heparan sulphate) remains to be investigated.

## 5. CRISPR as the Future of Diagnostic Screening

Current diagnostic methods, such as qPCR, have been proven to be highly effective in detecting target pathogens in patient samples; however, the current COVID-19 pandemic has highlighted several issues regarding turnaround time, reagent availability, cost, and access to equipment. As such, there has been a significant effort to develop novel SARS-CoV-2 diagnostics by exploiting the promiscuous cleaving capacity of type V (*Cas12*) and type VI (*Cas13)* CRISPR systems. CRISPR as a diagnostic tool has been shown to have significant advantages in that they are rapid, minimise reagents, more cost effective, time-efficient, and are easily field-deployable (i.e., to developing nations) with two platforms, DETECTR and SHERLOCK now in the development phase.

**Figure 3:**
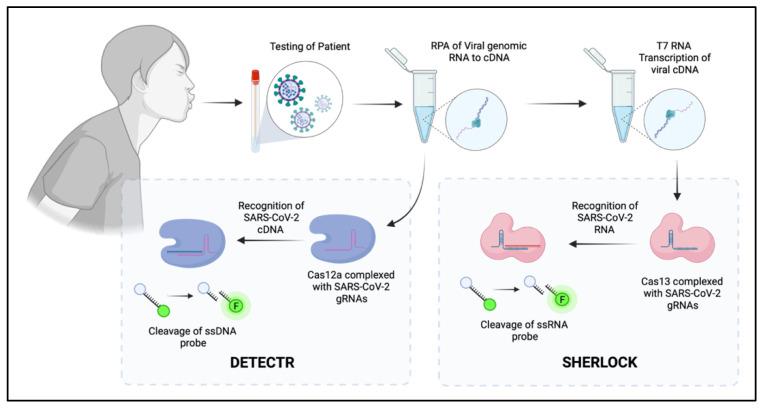
Summary of the process required for the DETECTR and SHERLOCK CRISPR diagnostic systems, which utilise the collateral cleavage capacity of both *Cas12* and *Cas13*.

SHERLOCK (Specific High Sensitivity Enzymatic Report unlocking), as shown in Figure 3, utilises *Cas13* and crRNAs that specifically target ssRNA and are therefore highly amenable to probing for RNA viral genomes in clinical samples [39,43,49]. Production and amplification of viral genomes are achieved through cDNA synthesis using Recombinase Polymerase Amplification (RPA) [143,144,145] that is coupled to a T7 polymerase reaction for RNA production. Within this reaction, a ssRNA-quenched probe is present. If the genome of the target pathogen is recognised by the crRNA, the ssRNA probe is cleaved non-specifically by the activated *Cas13*. This will produce a quantifiable signal that indicates the presence of the target. The DETECTR (DNA Endonuclease—Targeted CRISPR Trans Reporter) platform is in principle, like SHERLOCK as also shown in Figure 3; however, it is uses *Cas12* which is activated upon recognition of a target DNA. Diagnostics to identify DNA viral genomes can be directly used, but for RNA viruses’ incorporation of a RPA step is required for cDNA generation. Activation of *Cas12* by recognition of viral DNA results in the quantifiable cleavage of a ssDNA-quenched probe.

SHERLOCK and DETECTR have successfully been validated to detect SARS-CoV-2 [40,51,146,147] Ebola Virus [148], Lymphocytic Choriomeningitis Virus (LCMV), Influenza A Virus (IAV) and Vesicular Stomatitis Virus (VSV) [50]. Development of readout methodologies varies, spanning from fluorescence-based assays, that can also be implemented in multiplex high-throughput screening platforms [48], or as inexpensive, field-deployable lateral flow strip assays. Although not yet approved for diagnostics, CRISPR has the potential to revitalise health care for monitoring and detection of infectious viral diseases.

## 6. Conclusions

The adaptation of CRISPR has revolutionized gene editing, allowing advances in screening and diagnostic. This has vastly increased our understanding of the dynamic virus–host relationship and will continue to provide insight into similarities and differences in replications strategies, mechanisms of disease and immune evasion. In the future, this could allow for development of anti-viral therapeutics. Further developments in CRISPR technology targeting DNA and RNA will minimise off-target effects and allow for easy RNA editing to provide novel insights into viral replication and the effects on their host.

## Figures and Tables

**Table 2:** Summary of Human Genome-wide CRISPR Screens Identifying Critical Host Anti-Viral and Pro-Viral Factors.

Virus	CRISPR Screen	Cell Type	Host Factor Class	Top Candidates	Reference
Influenza A PR8	KO	A549	Pro-viral	WDR7, CCDC115, TMEM199, SLC35A1	[67]
Influenza A H5N1	KO	A549	Pro-Viral	SLC35A1, GDF11, IRX3, C2CD4C, TRIM23, PIGN, ACADSB, GRAMD2	[68]
Influenza A PR8	Activator	A549	Anti-Viral	B4GALNT2	[69]
Zika Virus PRVABC59 and MR766	KO	iPSC differentiated into NPC	Pro-Viral	WDR7, EMC1, EMC2, EMC4, ATP6V1A, MMGT1, TM9SF2, EXT2 EMC	[70]
Zika Virus MR766	KO	HeLa	Pro-Viral	AXL, EMC, MMGT1, SSR3, STT3A, WDR7, RABGEF1	[71]
Zika Virus MR766	KO	GSC	Pro-Viral	SSR3, STT3A, MMGT1, SSR2, TMEM41B, OXGR1, EMC, OST4	[72]
Zika Virus MR766	Activator	Huh7.5	Anti-Viral	IFI6, ISG20, ZCCHC6, IFN-λ2, IRF1, MAVS, TRIM25	[73]
Dengue Virus 16681	KO	Huh7.5	Pro-Viral	SSR1-3, ERAD Pathway (EMC), MMGT1, AGT1, STT3B, RPN2, STT3A, OST4	[8]
Dengue Virus Jamaican	KO	HAP1	Pro-Viral	SLC35B2, PAPSS1, B4GALT7, EXT2, STT3A, B3GAT3, DPM1, DPM3	[74]
Hepatitis C Virus JFH-1	KO	Huh7.5	Pro-Viral	CLDN1, OCLN, CD81, PPIA, RFK, FLAD1, ELAVL1, SRRD, ANKRD49, ZFB1	[8]
Human Immunodeficiency Virus	KO	THP-1	Pro-Viral	IFNAR1, IRF9, STAT1, STAT2, ZC3HAV1, TRIM25, N4BP1	[75,76]
Human Immunodeficiency Virus	KO	GXR	Pro-Viral	CD4, CCR5, ALCAM, SLC35B2, TPST2	[77]
Human Immunodeficiency Virus	KO	Jurkat T Cells	Pro-Viral (latency)	ZNF304, ARL16, ATF1, CGREF1, USMG5	[76]
West Nile Virus B596	KO	293T	Pro-Viral	SPCS1, SPCS3, EMC, OST complex (STT3A), TRAP complex, SEL1L, HRD1	[7]
West Nile Virus NY2000	KO	293T	Pro-Viral	STT3A, SEC63, SEC61B, OSTC, SPCS1, SPCS3, SERP1, EMC6, SEL1L, HSPA13, OST4, EMC4	[9]
Ebola Virus Mayinga	KO	Huh7.5	Pro-Viral	GNPTAB, NPC1, SPNS1, SLC30A1, VPS16, VPS33A and VPS18	[78]
Epstein–Barr Virus B95.8	KO	B Cell Lymphocytes	Pro-Viral	CD19, CD81, cFLIP, BATF, IRF4 and IRF2	[79]
Yellow Fever Virus YFV-17D	KO	Huh7.5	Pro-Viral	IFI6, BiP, IFN pathway (IFNAR, STAT2, JAK1), HSPA5	[80]
Murine Norovirus MNoV^CW3^ MNoV ^CR6^	Activator	HeLa	Anti-Viral	TRIM7, HOXC11, MX1, DDX60, PITX1	[81]
Murine Norovirus MNoV^CW3^ MNoV ^CR6^	KO	HeLa	Pro-Viral	CD300LF, G3BP1, KMT2D, CD300LH	[82]
Hepatitis A Virus HM175/18f	KO	Huh7.5	Pro-Viral	SLC35A1, ZCCHC14, EIF4B, PTBP1, PDAP1, SCAP, A1CF, FXR1, UFM1, PAPD7, PAPD5, UGCG, ST3GAL5	[83]
SARS-CoV-2 HCoV-229E	KO	Huh7	Pro-Viral	SARS-CoV-2: TMEM41B, TMEM106B, KRT19, AHCYL1, PTDSS1, OSBPL9, GLUD1, DTD1, EXT1, ACE2 HCoV-229E: ANPEP, TMEM41B, PIK3C3, NUFIP2	[84]
SARS-CoV-2	KO	Calu-3	Pro-Viral	AP1G1, ACE2, CHUK, TMPRSS2, AP1B1, RIPK4, ROCK1, AP1M2	[85]
SARS-CoV-2	Activator	Calu-3	Anti-Viral	TEAD3, MUC21, MUC4, MUC1, CPNE3, SPDEF, LY6E, JDP2, CCNE1, ZNF275	[85]
SARS-CoV-2	KO	A549	Pro-Viral	ACE2, ACTR2, ARPC3, ARPC4, RAB7A, CTSL, Retromer Complex, Commander Complex, PIK3C3, SPEN, SLTM, DPM3, ERMP1, PPID, CHST14	[86]
SARS-CoV-2 HCoV-229E HCoV-OC43	KO	Huh7.5	Pro-Viral	SARS-CoV-2: TMEM106B, VAC14, SCAP, ACE2, EXT1, PCDH19, MBTPS2 HCoV-229E: ANPEP, TMEM41B, VPS11, -16, -18, RAB7A, PIK3C3, GPR89A, GPR89B HCoV-OC43: B3GALT6, SLC35B2, EXT1, EXT2, B3GAT3, B4GALT7, FAM20B	[87]
SARS-COV-2 HCoV-229E HCoV-NL63 HCoV-OC43	KO	Huh7.5	Pro-Viral	SARS-CoV-2: TMEM41B, DHX36, EXTL3, EXT1, EXT2, ACE2, MBTPS2, SCAP, TMEM106B, VAC14, SLC35B2 HCoV-229E: ANPEP, TMEM41B, PIK3C3, VPS11, RAB7A HCoV-NL63: CDX2, ACE2, NRIP1, SMAD4, BMPR1A, EP300, KMT2B, SETDB1, AVCR1, KDM6A HCoV-OC43: B3GAT3, EXT1, EXT2, SLC35B2, B4GALT7, RAB7A, TM9SF3, XYLT2, SCAP, MBTPS1, NDST1, TMEM41B	[88]
SARS-CoV-2 MERS-CoV HKU5-SARS-CoV-1-S	KO	Vero	Pro-Viral	SARS-CoV-2: ACE2, CTSL, ARID1A, KDM6A, SMARCC1, HMGB1, SMARCA4 MERS-CoV: DPP4, AXIN1, TMEM41B, MTF1, CTSL, ARID1A, KDM6A HKU5-SARS-CoV-1-S: CTSL, ACE2, SMARCA4, JMJD6, PHIP, KDM6A	[89]
SARS-CoV-2	KO	A549	Pro-Viral	Commander complex, Retromer Complex, ACE2, WDR81, ARPC4, NPC1, CTSL	[90]
SARS-CoV-2	KO	Calu-3	Pro-Viral	AP1G1, ACE2, TMPRSS2, KMT2C, ARID2, KDM6A	[91]
SARS-CoV-2	Activator	Calu-3	Anti-Viral	LY6E, MUC21, TEAD3, PLAGL1, MUC4, MUC1, JADE3	[91]
MERS-CoV	KO	Huh7	Pro-Viral	DPP4, HNF1A, PTBP1, CLCN5, PCTP, OR9K2	[92]
HCoV-229E	KO	Huh7	Pro-Viral	VMP1, ANPEP, PHGDH, TMEM41B, LAMB3, BCL21	[92]
HCoV-229E	KO	Huh7	Pro-Viral	ANPEP, TMEM41B	[93]

**Table 3:** Summary of commercially available CRISPRa systems utilised *dCas9* that are already widely used for genome-wide screening.

System		Components
CRISPR VPR	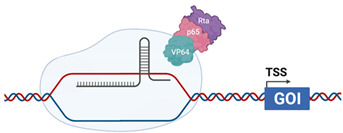	dCas9 is fused with the transcriptional activators VP64 (HSV), p65 (cellular) and Rta (EBV) [124]
CRISPRaSAM	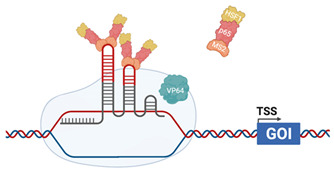	The sgRNA incorporates MS2 RNA aptamers in the stem–loop Helper complex, composed of MS2 coat protein, p65 (cellular) and Heat Shock Factor 1 (HSF1-cellular) dCas9 is fused with VP64 (HSV). Helper complex binds to the MS2 RNA aptamers [125]
CRISPRaSunTag	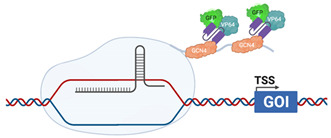	dCas9 is fused with a GCN4 repeating polypeptide, “SunTag” ScFv, fused with VP64 (HSV) and GFP, are raised against GCN4, allow recruitment of additional transcriptional activators to the promoter [126]

## Data Availability

All data contained within this review are accessible using publicly searchable article databases.
